# Contributions of *NFKB1* ‐94insertion/deletion ATTG polymorphism to the susceptibility of gastrointestinal cancers: A meta‐analysis

**DOI:** 10.1111/jcmm.17004

**Published:** 2021-10-21

**Authors:** Hanqiang Wu, Jianrong Liang

**Affiliations:** ^1^ Department of Gastrointestinal Surgery The First People’s Hospital of Zhaoqing Zhaoqing China

**Keywords:** gastrointestinal cancers, meta‐analysis, *NF‐κB1*, SNPs, susceptibility

## Abstract

Nuclear factor‐kappa B1 (NF‐κB1), a pleiotropic transcription factor, functions as a critical contributor to tumorigenesis. Growing numbers of case‐control studies were carried out to analyse the potential contribution of *NF‐κB1* gene variants to gastrointestinal cancer risk, yet remains conflicting conclusions. Therefore, we conducted this most up‐to‐date meta‐analysis to evaluate the relationship between *NF‐κB1* gene insertion (I)/deletion (D) polymorphism, namely −94ins/delATTG or rs28362491, and the susceptibility to gastrointestinal cancers. We searched PubMed, EMBASE and MEDLINE databases updated in April 2021 for relevant studies. Meta‐analysis was carried out by software Stata11.0. The quantification of the relationship was determined by computing the combined odds ratios (ORs) and their corresponding 95% confidence intervals (CIs). Sensitivity analysis, the funnel plot and Begg's rank correlation test were also applied. Our findings indicate that −94ins/delATTG polymorphism could not significantly impact the susceptibility to gastrointestinal cancers. Under any five genetic models, −94ins/delATTG polymorphism was not remarkedly linked to the risk of colorectal, gastric and oesophageal cancer, respectively. The significant role of −94ins/delATTG was only observed in some certain subgroups. Findings here suggest that *NF‐κB1* gene −94ins/delATTG polymorphism may not predispose to gastrointestinal cancer susceptibility.

## 1 INTRODUCTION

An estimated 4.8 million new cases of gastrointestinal cancers and 3.4 million related deaths occurred worldwide in 2018.^1^ Gastrointestinal cancers account for 26% of the global cancer incidence and 35% of all cancer‐related deaths.^1^ Gastrointestinal cancers mainly include oesophageal, gastric and colorectal cancers.^2^ Most of the gastrointestinal cancers were diagnosed at a middle or advanced stage. This situation is the major obstacle to the effective treatment of gastrointestinal cancers.^3^, ^4^ Therefore, identifying early diagnosis markers for gastrointestinal cancers is of great significance to the treatment of gastrointestinal cancers.

Gastrointestinal cancers are multifactorial diseases caused by complex interactions between genetic and environmental factors.^5^, ^6^, ^7^ More than 50% of all gastrointestinal cancers are caused by environmental risk factors, including infection, alcohol consumption, tobacco smoking and over obesity. Apart from the environmental factors, genetic variations are also implicated in the onset and outcome of gastrointestinal cancers.^8^, ^9^, ^10^, ^11^ Single nucleotide polymorphisms (SNPs), the most common and effective variant type, are significantly associated with cancer susceptibility.^12^, ^13^, ^14^

NF‐κB, short for nuclear factor kappa B, is a pluripotent and critical dimer transcription factor. NF‐κB orchestrates multiple physiological and pathological processes, particularly in cell survival, differentiation, inflammation and carcinogenesis.^15^, ^16^, ^17^, ^18^, ^19^, ^20^, ^21^, ^22^, ^23^ It was originally discovered by Sen and Baltimore in 1986.^24^ NF‐κB family is consisted of 5 different protein subunits, including NF‐κB1 (p50/p105), NF‐κB2 (p52/p100), RelA (p65), c‐Rel and RelB, in mammals.^25^ NF‐κB expression is strictly regulated in normal cells, but it is generally overexpressed in many cancer cells.^26^ Upregulation of NF‐κB has been observed in several types of cancer, including hepatocellular carcinogenesis,^27^, ^28^ colon cancer,^29^ breast cancer,^30^ ovarian cancer^31^ and glioma cancer.^32^ Multiple number of *NFKB1* gene SNPs were investigated in the implication of cancer. Among them, the rs28362491, namely the −94insertion/deletion ATTG polymorphism, ranks the most intensively investigated SNP.^33^, ^34^ The deletion of ATTG bases prevents or reduces the binding to nuclear proteins and results in decreased transcript levels of the *NFKB1* gene, thus influencing the stability of mRNA and efficiency of regulating translation. Research regarding *NFKB1* gene −94insertion/deletion ATTG polymorphism on its association with gastrointestinal cancer risk was widely performed. However, the conclusions are still contradictory and inconsistent, partly attributed to the underpower and bias of independent studies, especially for small cohorts. Therefore, the exact association between *NFKB1* −94insertion/deletion ATTG variant and risk of gastrointestinal cancer awaits to be determined. Here, a renewed meta‐analysis with all potential studies performed before April 2021 was analysed to acquire a clearer impact of *NFKB1* −94insertion/deletion ATTG polymorphism on gastrointestinal cancer susceptibility.

## 2 MATERIALS AND METHODS

### 2.1 Search strategy

We reported this meta‐analysis in accordance with the Preferred Reporting Items for Systematic Reviews and Meta‐analyses (PRISMA) and Meta‐analysis of Observational Studies in Epidemiology (MOOSE) reporting guidelines.

### 2.2 Publication selection

We applied an all‐sided literature retrieval using EMBASE, PubMed and MEDLINE up to April 2021. We used the following key words to carry out this procedure: (1) *NFKB1* or NF‐κB1 or nuclear factor kappa B1; (2) −94insertion/deletion ATTG or rs28362491 or SNPs or polymorphisms or polymorphism or variants; and (3) colorectal cancer or gastric cancer or gastrointestinal cancers or oesophageal cancer. To identify all the available studies, we also arranged two authors to screen eligible publications by hand‐searching the references of included publications.

### 2.3 Eligibility criteria

Publications are required to meet the following criteria for inclusion: (1) evaluating the relationship between *NFKB1* rs28362491 and gastrointestinal cancer risk; (2) case‐control study; and (3) enough data to calculate odds ratios (ORs) and corresponding 95% confidence intervals (CIs). Editorials, meta‐analyses, reviews, research on animals and repetitively published articles were excluded.

### 2.4 Data extraction

We arranged two researchers (Hanqiang Wu and Jianrong Liang) to acquire the data independently by adopting the unified data table. The authors acquired the following information from all the studies: surname of the first author, source of control, year of publication, type of cancers, ethnicity of the study subject, numbers of cases and controls, genotyping method, and genotype of SNPs. If the extracted information is disputable, the authors would re‐check the references and make sure which extracted information is right. We assessed the methodologic quality of each study using the quality assessment criteria described by previous studies (Table S1). We judged the study quality to be high if the score was more than 9 points or otherwise to be low.

### 2.5 Statistical methods

We first test if the SNPs in the controls conformed to Hardy‐Weinberg equilibrium (HWE) by goodness‐of‐fit test. The association between *NFKB1* rs28362491 and gastrointestinal cancer risk was evaluated by identifying the genotype frequencies of all cases and controls. Odds ratio (OR) and 95% confidence interval (CI) were adopted to assess this relationship. The meta‐analysis assessed association by using five different genetic models: homozygote model (II, homozygous insertion [ins/ins] or wild‐type vs. DD, homozygous deletion [del/del]); heterozygote model (ID, heterozygous ins/del vs. DD); recessive model (II vs. ID/DD); dominant model (ID/II vs. DD); and allele contrast model (I vs. D). Stratification analyses were also performed by ethnicity, cancer type, source of control, score and HWE in controls, if applicable. We further analysed the heterogeneity among studies using Cochrane Q test. If *p* < 0.10 or I^2^ > 50% for the *Q* test, the random effects model (DerSimonian‐Laird method) was applied to conduct the analysis; if not, the fixed effects model (Mantel‐Haenszel method) was applied.^35^ The included studies were deleted one by one, and then, the left studies were recalculated for ORs and 95% CIs to determine the influence of each study on the total combined effect size (sensitivity analysis). Publication bias for the included literature was determined using the funnel plot and Begg's funnel plot. *p* value < 0.05 indicates significant finding. Trial sequential analysis (TSA) was performed as described by us previously. Briefly, after adopting a level of significance of 5% for type I error and of 30% for type II error, the required information size was calculated, and TSA monitoring boundaries were built. All statistical analysis was performed using STATA, version 11.0 (Stata Corporation, College Station, TX).

## 3 RESULTS

### 3.1 Literature retrieval results

**FIGURE 1:**
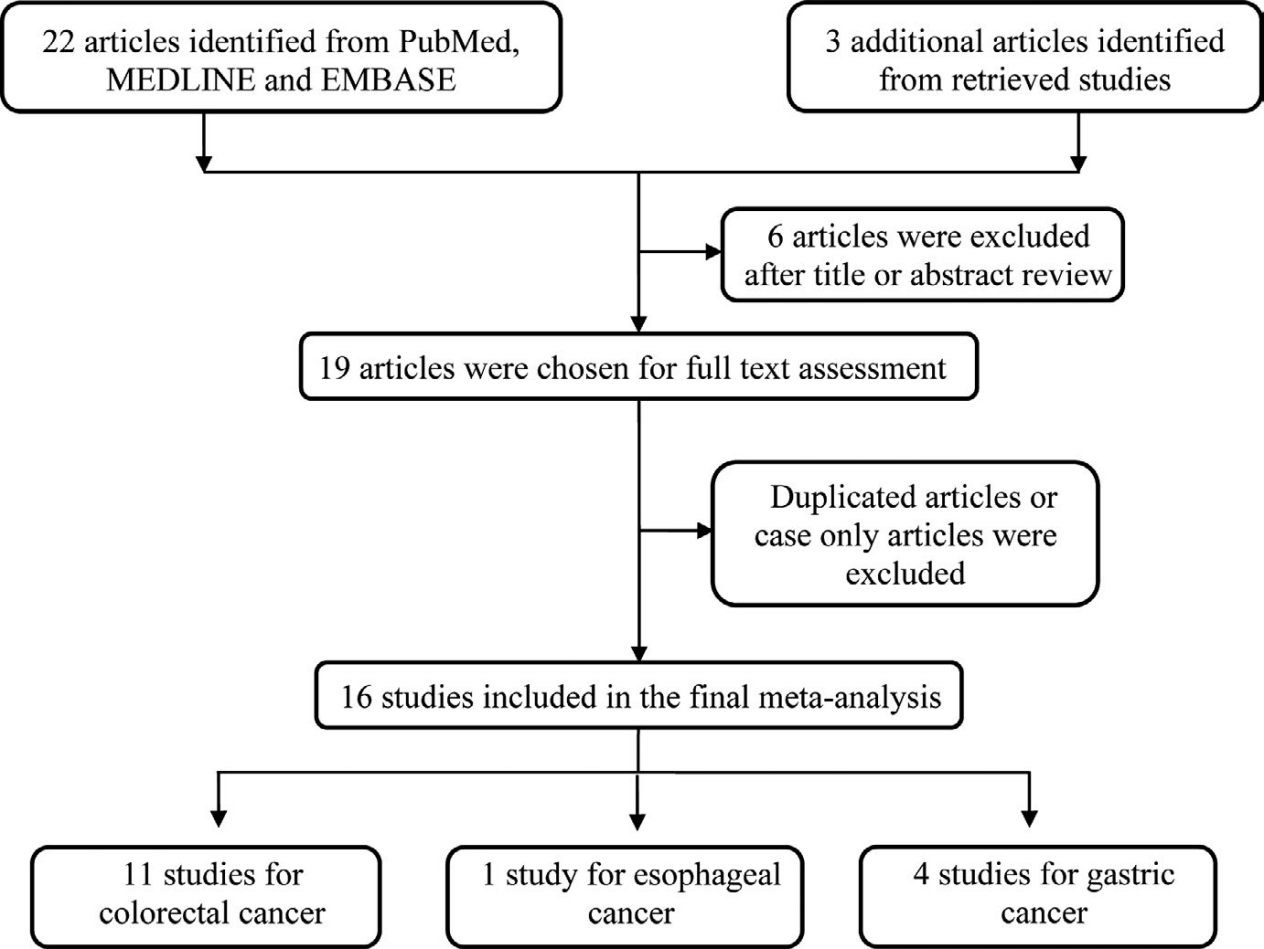
PRISMA flow chart illustrating results of the literature

The screening process of the current meta‐analysis was shown in Figure 1. We first identified 22 articles with potential relevance from PubMed, MEDLINE and EMBASE. We then arranged two authors to identify whether there exist additional articles from the retrieved studies. Three articles were further identified. After careful review, we total identified 16 studies for final analysis.^33^, ^36^, ^37^, ^38^, ^39^, ^40^, ^41^, ^42^, ^43^, ^44^, ^45^, ^46^, ^47^, ^48^

### 3.2 Studies characteristics

Among the 16 included studies (Table 1), 11 were reported on colorectal cancer, 4 on gastric cancer and 1 on oesophageal cancer. Nine were in Asian populations, 8 in Caucasian populations and 2 in Asian populations. For sources of control, 12 were hospital based and 4 were population based. For quality score, there were 8 publications of low quality and 8 studies of high quality. The SNPs in the control groups of 10 studies complied with HWE, while 6 deviated.

**TABLE 1:** Characteristics of studies included in the current meta‐analysis

Surname	Year	Cancer type	Country	Ethnicity	Control Source	Genotype method	Case				Control				Score	HWE
							II	ID	DD	All	II	ID	DD	All		
Riemann	2006	Colorectal	Germany	Caucasian	HB	Pyrosequencing	54	58	27	139	118	141	48	307	9	0.586
Lewander	2007	Colorectal	China	Asian	HB	PCR‐RFLP	50	101	42	193	113	266	79	458	9	<0.001
Lewander	2007	Colorectal	Sweden	Caucasian	HB	PCR‐RFLP	63	323	81	467	116	256	67	439	10	<0.001
Lo	2009	Gastric	China	Asian	HB	PCR	62	89	31	182	20	62	34	116	7	0.361
Vibeke	2010	Colorectal	Denmark	Caucasian	PB	TaqMan	121	195	62	378	307	347	102	756	11	0.801
Song	2011	Colorectal	China	Asian	HB	PCR‐RFLP	363	500	138	1,001	297	522	186	1,005	14	0.102
Ungerback	2012	Colorectal	Sweden	Caucasian	HB	TaqMan	114	187	43	344	256	270	96	622	8	0.079
Arisawa	2013	Gastric	Japan	Asian	PB	PCR‐SSCP	172	239	68	479	342	435	103	880	11	0.046
Umar	2013	Oesophageal	India	Asian	HB	PCR	131	132	27	290	160	129	22	311	10	0.561
Mohd	2013	Colorectal	Malaysia	Asian	HB	PCR‐RFLP	35	127	75	237	16	138	83	237	9	<0.001
Hua	2014	Gastric	China	Asian	HB	MassARRAY	92	182	127	401	120	230	83	433	9	0.144
Kopp	2015	Colorectal	Denmark	Caucasian	PB	KASP	320	449	146	915	679	787	253	1,719	11	0.311
Mohamed	2017	Colorectal	Egypt	Caucasian	HB	PCR‐RFLP	30	56	14	100	4	58	23	85	9	<0.001
Giovanna	2017	Gastric	Brazil	Caucasian	HB	PCR	15	71	34	120	110	246	117	473	10	0.379
Giovanna	2017	Colorectal	Brazil	Caucasian	HB	PCR	5	42	16	63	110	246	117	473	10	0.379
Imene	2019	Colorectal	Algeria	Caucasian	PB	TaqMan	54	56	17	127	68	57	24	149	9	0.048

Abbreviations: HB, hospital based; HWE, Hardy‐Weinberg equilibrium; KASP, kompetitive allele specific PCR; PB, population based; PCR‐RFLP, polymerase chain reaction‐restriction fragment length polymorphism; PCR‐SSCP, polymerase chain reaction‐single strand conformation polymorphism.

### 3.3 Quantitative analysis

**FIGURE 2:**
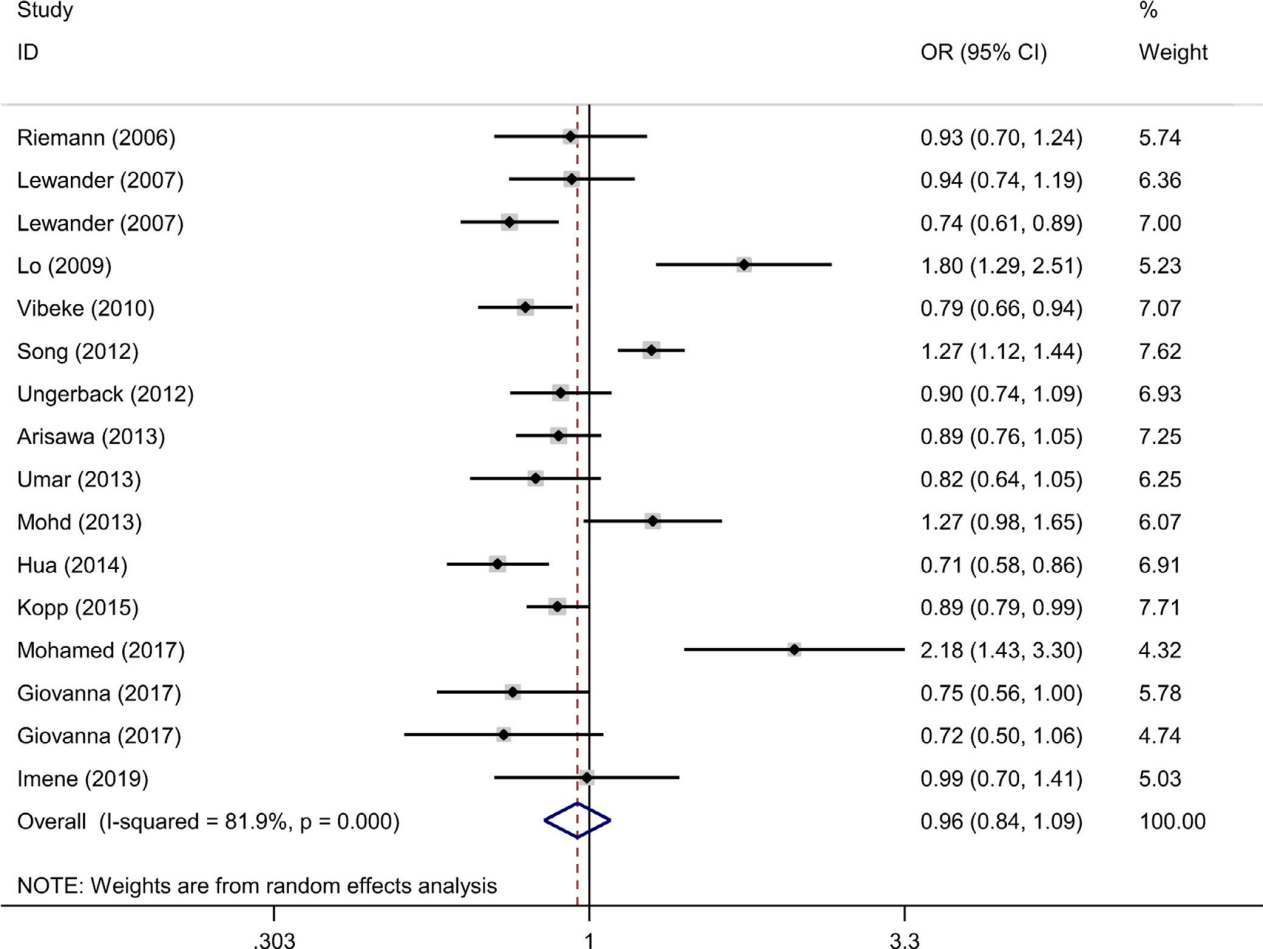
Representative forest plots for the correlation between the *NFKB1* −94ins/delATTG polymorphism and gastrointestinal cancer susceptibility. The horizontal lines represent the study‐specific ORs and 95% CIs

**FIGURE 3:**
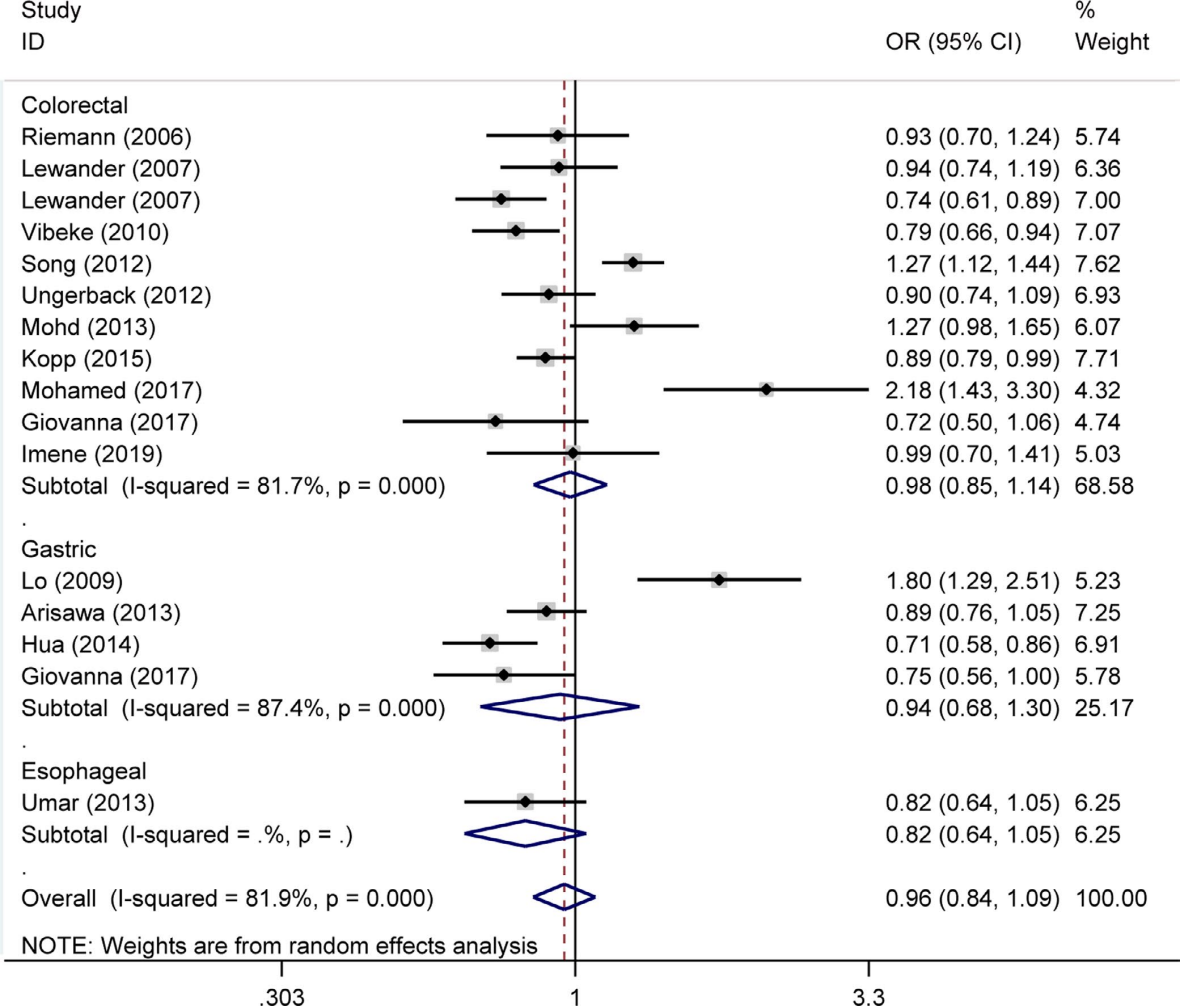
Representative forest plots for the correlation between the *NFKB1* −94ins/delATTG polymorphism and respective oesophageal, gastric and colorectal cancers susceptibility. The horizontal lines represent the study‐specific ORs and 95% CIs

The detailed results of the meta‐analysis were presented in Table 2 and Figures 2 and 3. The pooled analyses indicated that negative association was detected between the *NFKB1* −94ins/delATTG polymorphism and overall gastrointestinal cancer susceptibility under all 5 genetic models (II vs. DD: OR = 0.94, 95% CI = 0.70–1.26; ID vs. DD: OR = 0.99, 95% CI = 0.85–1.16; II vs. ID/DD: OR = 0.92, 95% CI = 0.74–1.14; ID/II vs. DD: OR = 0.97, 95% CI = 0.81–1.16; and I vs. D: OR = 0.96, 95% CI = 0.84–1.09). In cancer type subgroup analysis, *NFKB1* −94ins/delATTG polymorphism still failed to impact the susceptibility in subgroup of colorectal cancer, gastric cancer and oesophageal cancer. Subgroup analysis of ethnicity indicated that significant decreased cancer risk was detected in Caucasians (II vs. ID/DD: OR = 0.74, 95% CI = 0.56–0.98), but not in Asians. Further stratification analysis by source of control revealed that studies conducted as population base protect from cancer risk (II vs. DD: OR = 0.78, 95% CI = 0.66–0.93; II vs. ID/DD: OR = 0.81, 95% CI = 0.72–0.91; and I vs. D: OR = 0.87, 95% CI = 0.80–0.94). Moreover, score subgroup indicated that studies >9 were linked to decreased cancer risk (II vs. DD: OR = 0.70, 95% CI = 0.49–0.99; II vs. ID/DD: OR = 0.72, 95% CI = 0.55–0.94; and I vs. D: OR = 0.87, 95% CI = 0.74–1.00). Further stratification of HWE analysis in controls demonstrated that no significant association was detected in subgroup of both HWE ≤ 0.05 and HWE > 0.05.

**TABLE 2:** Meta‐analysis of the association between *NFKB1* −94Ins/Del (rs28362491) polymorphism and gastrointestinal cancer risk

Variables	No. of studies	Homozygous		Heterozygous		Recessive		Dominant		Allele	
		II vs. DD		ID vs. DD		II vs. ID/DD		ID/II vs. DD		I vs. D	
		OR (95% CI)	*p* ^het^	OR (95% CI)	*p* ^het^	OR (95% CI)	*p* ^het^	OR (95% CI)	*p* ^het^	OR (95% CI)	*p* ^het^
All^a^	16	0.94 (0.70–1.26)	<0.001	0.99 (0.85–1.16)	0.005	0.92 (0.74–1.14)	<0.001	0.97 (0.81–1.16)	<0.001	0.96 (0.84–1.09)	<0.001
Cancer type											
Colorectal	11	1.01 (0.71–1.43)	0.001	1.07 (0.93–1.23)	0.2111	0.94 (0.71–1.25)	<0.001	1.03 (0.87–1.23)	0.021	0.98 (0.85–1.14)	<0.001
Gastric	4	0.85 (0.43–1.71)	<0.001	0.87 (0.57–1.33)	0.006	0.93 (0.58–1.45)	0.001	0.88 (0.54–1.43)	<0.001	0.94 (0.68–1.30)	<0.001
Oesophageal	1	0.67 (0.36–1.23)	‐	0.83 (0.45–1.54)	‐	0.78 (0.56–1.07)	‐	0.74 (0.41–1.33)	‐	0.82 (0.64–1.05)	‐
Ethnicity											
Caucasians	9	0.80 (0.56–1.13)	<0.001	1.06 (0.93–1.22)	0.446	**0.74 (0.56–0.98)**	<0.001	0.96 (0.82–1.12)	0.219	0.89(0.77–1.02)	0.001
Asians	7	1.13 (0.70–1.82)	<0.001	0.90 (0.67–1.21)	0.004	1.16 (0.88–1.53)	<0.001	0.95 (0.67–1.35)	<0.001	1.03 (0.84–1.28)	<0.001
Source of control											
HB	12	1.02 (0.67–1.55)	<0.001	1.01 (0.82–1.26)	0.001	0.99 (0.72–1.37)	<0.001	1.01 (0.79–1.28)	<0.001	0.99 (0.83–1.19)	<0.001
PB	4	**0.78 (0.66–0.93)**	0.562	0.95 (0.81–1.13)	0.625	**0.81 (0.72–0.91)**	0.539	0.87 (0.75–1.02)	0.629	**0.87 (0.80–0.94)**	0.579
Score											
≤9	8	1.40 (0.82–2.38)	<0.001	1.01 (0.72–1.41)	<0.001	1.30 (0.89–1.90)	<0.001	1.07 (0.75–1.53)	<0.001	1.10 (0.88–1.39)	<0.001
>9	8	**0.70 (0.49–0.99)**	<0.001	1.03 (0.91–1.16)	0.554	**0.72 (0.55–0.94)**	<0.001	0.97 (0.81–1.15)	0.046	**0.86 (0.74–1.00)**	<0.001
HWE											
>0.05	10	0.84 (0.60–1.18)	<0.001	1.004 (0.81–1.25)	0.001	0.85 (0.67–1.07)	<0.001	0.95 (0.74–1.21)	<0.001	0.91 (0.78–1.07)	<0.001
≤0.05	6	1.24 (0.67–2.31)	<0.001	0.96 (0.80–1.16)	0.372	1.19 (0.71–2.00)	<0.001	0.99 (0.77–1.27)	0.086	1.05 (0.83–1.33)	<0.001

Note

The bold values indicate significant results.

Abbreviations: HB, hospital based; Het, heterogeneity; PB, population based.

^a^ All the studies included.

### 3.4 Sensitivity analysis

**FIGURE 4:**
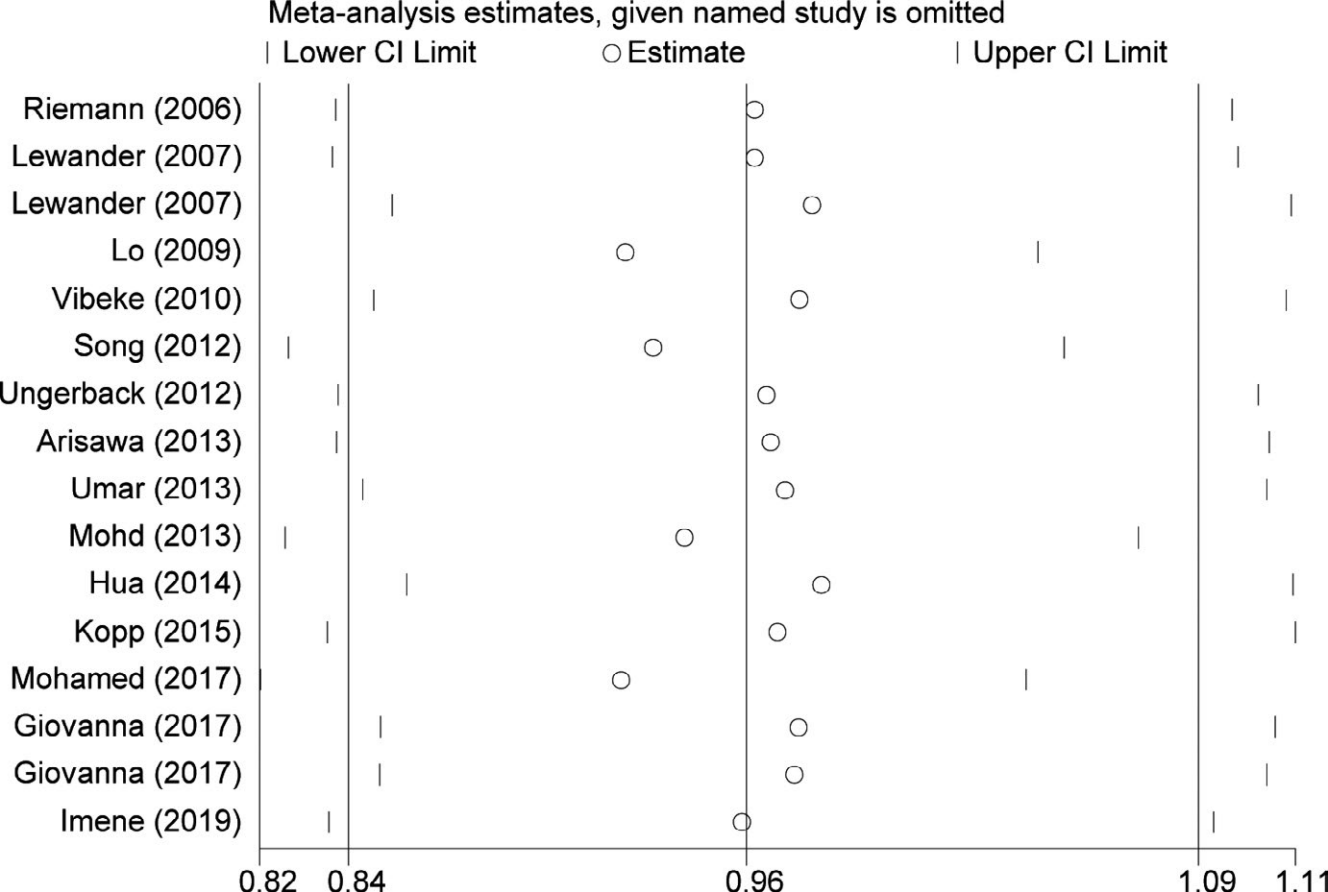
Sensitivity analysis of the association between *NFKB1* −94ins/delATTG polymorphism and gastrointestinal cancer susceptibility. Each point represents the recalculated OR after deleting a separate study

Here, we also carried out a sensitivity analysis by gradually deleting the included studies in a one‐by‐one manner. The no statistical fluctuation of the pooled OR value suggested that the analytic results were reliable and stable (Figure 4).

### 3.5 Publication bias

**FIGURE 5:**
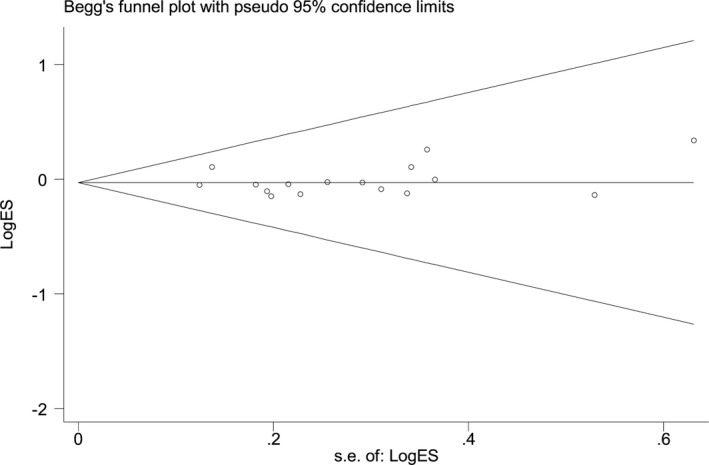
Funnel plot analysis of publication bias for *NFKB1* −94ins/delATTG polymorphism. Each point represents a separate study

Begg's funnel plot did not have significant asymmetry (Figure 5). Statistical evidence of Egger's test also could not identify obvious publication bias for all of the polymorphisms.

### 3.6 Trial sequential analysis

**FIGURE 6:**
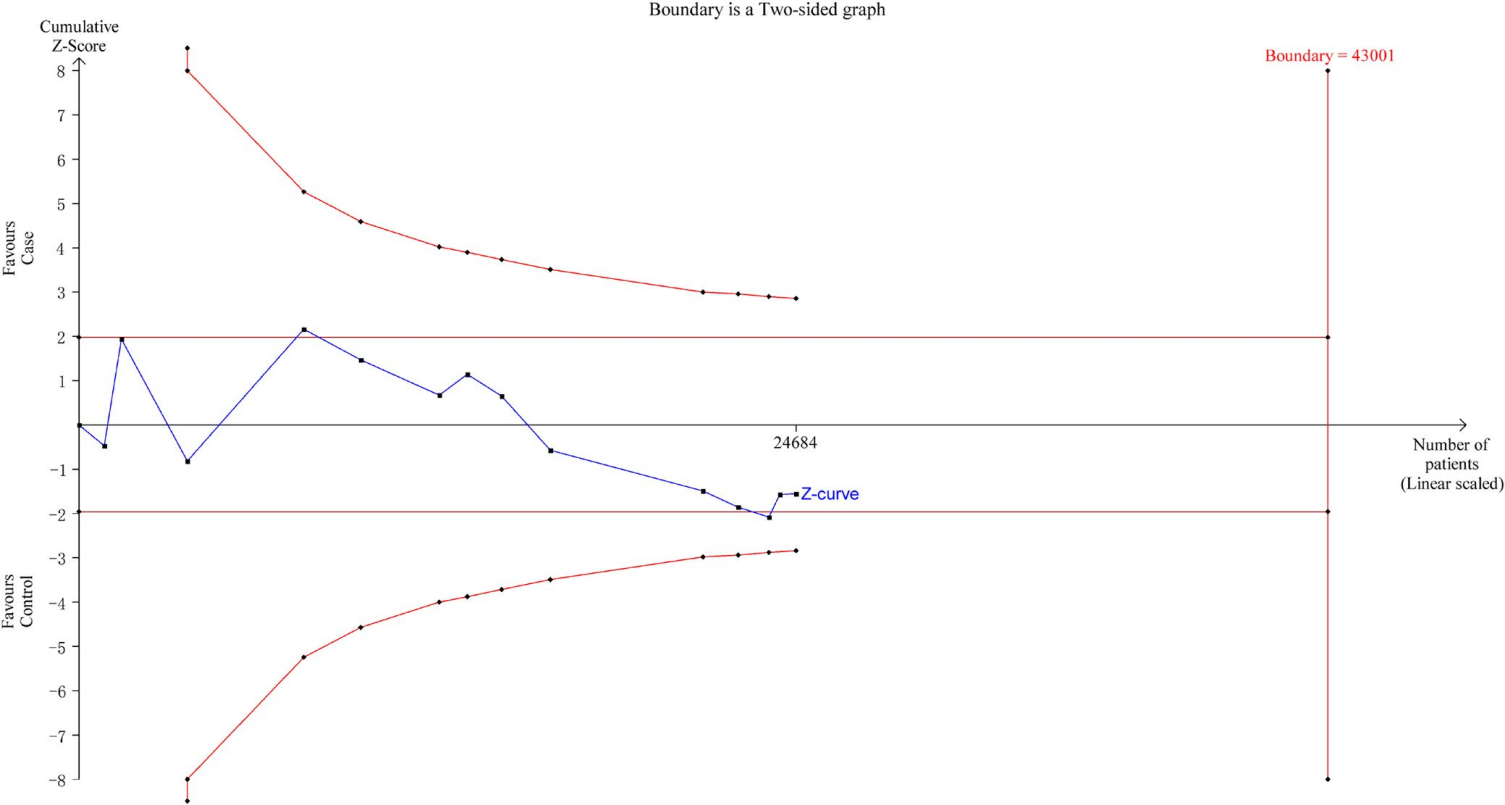
Trial sequential analysis for *NFKB1* −94ins/delATTG polymorphism under the allele contrast model

To minimize random errors and strengthen the robustness of our conclusions, we performed TSA (Figure 6). This analysis showed that the cumulative z‐curve did cross the trial sequential monitoring boundary, suggesting that further evidence is needed to verify the conclusions.

## 4 DISCUSSION

In the current meta‐analysis, we comprehensively extract information from available epidemiology studies to assess the association between *NFKB1* gene −94ins/delATTG polymorphism and gastrointestinal cancer risk. Our findings indicate that *NFKB1* gene −94ins/delATTG polymorphism could not modify gastrointestinal cancer susceptibility. Of note, this is the first meta‐analysis performed by far on *NFKB1* gene −94ins/delATTG polymorphism and gastrointestinal cancer susceptibility.

Growing evidence has pointed to the involvement of *NFKB1* −94ins/delATTG polymorphism analysed here (rs28362491) in cancer susceptibility. Song et al.^40^ observed that the *NFKB1* −94ins/delATTG polymorphism could enhance the susceptibility of colorectal cancer in a Southern Chinese population. However, role of *NFKB1* −94ins/delATTG polymorphism in specific cancer is contradictory, namely a decreased cancer susceptibility or a null association. To solve this controversy, several meta‐analyses have been conducted. The first meta‐analysis was carried out in 2011 by Zou et al.^49^ Their study incorporated 2743 cases and 2195 controls by including eleven studies. They failed to detect any relationship between the −94ins/delATTG SNP and risk of overall cancer. However, subgroup analysis identified an ethno‐specific association; the D allele could decrease the risk of cancer in Asians, but confer to a higher risk in Caucasians. In a meta‐analysis updated to July 2016 involving 18,299 cases and 23,484 controls from 50 case‐control studies, Fu et al. y^50^ identified that the *NFKB1* −94ins/delATTG polymorphism protects from getting overall cancer in the homozygote model; heterozygote model; dominant model; and allele contrast. Stratified and subgroup analyses indicated decreased susceptibility for prostate cancer, ovarian cancer, lung cancer, nasopharyngeal carcinoma and oral squamous cell carcinoma, and this association also is significant for Asians, especially Chinese subjects, in hospital‐based studies, and in studies with quality score <9. Of note, by far, no available GWAS has identified the significant relationship between *NFKB1* −94ins/delATTG polymorphisms and risk of gastrointestinal cancers.

Since then, several new relevant case‐control studies on gastrointestinal cancers have emerged. Therefore, we set as a pioneer to determine whether *NFKB1* −94ins/delATTG polymorphism impact risk of gastrointestinal cancers. However, *NFKB1* −94ins/delATTG polymorphism under any five genetic models has not enough ability to influence susceptibility to overall gastrointestinal cancer. Several merits existed in the current meta‐analysis. First, this is up to now the first and largest meta‐analysis regarding *NFKB1* gene −94ins/delATTG polymorphism and gastrointestinal cancers susceptibility. Second, in sensitivity analysis, relative stability and credibility of the results were achieved as no significant changes after deleting each study at a time. Third, there existed no obvious publication bias, indicating the reliability of conclusion. Some limitations in the current meta‐analysis should be acknowledged. First, the number of subjects in the included studies is relatively small, especially in stratified analyses, which might result in a lack of statistical power and prevent a meaningful analysis of the results. Second, the association strength was only evaluated by unadjusted estimates. Adjustment analysis including gene‐gene, gene‐environment factors were not carried out because of the lack of original data. Third, in the included studies, the population sources were generally limited to Caucasians and Asians. Thus, the conclusion here should be interpreted in caution in Africans.

## 5 CONCLUSION

In all, our finding has concluded that *NFKB1* gene −94ins/delATTG polymorphism may not predispose to gastrointestinal cancers susceptibility. More attention should be paid to several research directions in future studies. First, more high‐quality studies with expanded sample sizes are necessarily called to further identify the relationship of the *NFKB1* gene −94ins/delATTG polymorphism and gastrointestinal cancer risk, especially in Caucasian and African populations. Second, functional studies are needed to clarify the underlying mechanisms of *NFKB1* gene −94ins/delATTG in gastrointestinal cancers.

## CONFLICT OF INTEREST

The authors declare that there are no competing interests associated with the manuscript.

## AUTHOR CONTRIBUTIONS

**Hanqiang Wu:** Conceptualization (equal); Methodology (equal); Writing‐original draft (equal); Writing‐review & editing (equal). **Jianrong Liang:** Conceptualization (equal); Data curation (equal); Investigation (equal); Software (equal); Supervision (equal); Validation (equal); Writing‐original draft (equal); Writing‐review & editing (equal).

## Supporting information

Table S1

## Data Availability

The data sets used and/or analysed during the current study are available from the corresponding author on reasonable request.
