# Supplementary material for: Contributions of NFKB1 ‐94insertion/deletion ATTG polymorphism to the susceptibility of gastrointestinal cancers: A meta‐analysis
Source: J Cell Mol Med. 2021 Oct 21;25(22):10674–83. doi: 10.1111/jcmm.17004 (PMC8581328; doi:10.1111/jcmm.17004)
Supplement: Supplementary file 1 — Table S1 [file JCMM-25-10674-s001.doc]

| Supplemental Table S1. Score of quality assessment | |
| --- | --- |
| **Criteria** | **Score** |
| Representativeness of cases |  |
| Selected from population cancer registry | 2 |
| Selected from hospital | 1 |
| No method of selection described | 0 |
| Representativeness of controls |  |
| Population-based | 3 |
| Blood donors | 2 |
| Hospital-based | 1 |
| Not described | 0 |
| Ascertainment of cancer cases |  |
| Histopathologic confirmation | 2 |
| Patient medical record | 1 |
| Not described | 0 |
| Control selection |  |
| Controls matched with cases by age and sex | 2 |
| Controls matched with cases only by age or by sex | 1 |
| Not matched or not descried | 0 |
| Genotyping examination |  |
| Genotyping done blindly and quality control | 2 |
| Only genotyping done blindly or quality control | 1 |
| Unblinded and without quality control | 0 |
| Total sample size for both cases and controls |  |
| Larger than 1000 | 3 |
| Larger than 500, but less than 1000 | 2 |
| Larger than 200, but less than 500 | 1 |
| Less than 200 | 0 |
